# Supplementary material for: Shaped by the COVID-19 pandemic: Psychological responses from a subjective perspective–A longitudinal mixed-methods study across five European countries
Source: PLoS One. 2023 Apr 25;18(4):e0285078. doi: 10.1371/journal.pone.0285078 (PMC10128933; doi:10.1371/journal.pone.0285078)
Supplement: S1 Appendix — (PDF) [file pone.0285078.s001.pdf]

S1 Appendix. Detailed information on the methodology and participants' characteristics.

COVID-19 situation in the participating countries during the data assessment

S1 Table. COVID-19 situation at two timepoints of data collection in five countries.

**T1 (Baseline assessment)**					
**Countries**	**Austria**	**Croatia**	**Georgia**	**Greece**	**Portugal**
Recruitment period	27.06.2020 (week 26) – 22.09.2020 (week 39)	15.06.2020 (week 25) – 16.08.2020 (week 33)	03.07.2020 (week 27) – 13.10.2020 (week 42)	10.10.2020 (week 41) – 14.12.2020 (week 51)	29.07.2020 (week 31) – 04.11.2020 (week 45)
Duration of data collection	88 days ~ 13 weeks	63 days ~ 9 weeks	103 days ~ 15 weeks	66 days ~ 10 weeks	99 days ~ 15 weeks
**Stringency index**					
First week	50.00	54.63	57.93	50.46	61.97
Last week	37.04	35.19	60.85	84.26	70.24
Mean	38.59	44.98	57.53	71.49	61.59
Median	37.96	46.30	57.41	78.70	60.65
**Incidence**					
First week	4.04/1M	0.95/1M	0.83/1M	33.85/1M	19.91/1M
Last week	79.18/1M	23.05/1M	165.84/1M	102.56/1M	407.09/1M
Mean	24.74/1M	15.09/1M	26.83/1M	148.19/1M	92.68/1M
Median	14.02/1M	16.30/1M	3.32/1M	157.81/1M	57.39/1M
**Deaths**					
First week	0.26/1M	0.03/1M	0.03/1M	0.47/1M	0.32/1M
Last week	0.43/1M	0.24/1M	1.79/1M	7.91/1M	4.25/1M
Mean	0.19/1M	0.22/1M	0.17/1M	4.43/1M	0.83/1M
Median	0.18/1M	0.21/1M	0.00/1M	3.39/1M	0.40/1M
**T2 (Follow-up assessment)**					
**Countries**	**Austria**	**Croatia**	**Georgia**	**Greece**	**Portugal**

S1 Appendix. Detailed information on the methodology and participants' characteristics.

Recruitment period	13.7.2021 (week 28) – 08.10.2021 (week 40)	21.06.2021 (week 25) – 26.07.2021 (week 30)	22.07.2021 (week 29) – 25.10.2021 (week 43)	01.10.2021 (week 39) – 14.12.2021 (week 50)	31.08.2021 (week 35) – 25.11.2021 (week 47)
Duration of data collection	88 days ~ 13 weeks	36 days ~ 5 weeks	96 days ~ 14 weeks	75 days ~ 11 weeks	87 days ~ 13 weeks
**Stringency index**					
First week	48.98	39.29	25.00	71.43	56.48
Last week	46.46	31.84	47.22	73.07	40.74
Mean	50.11	35.19	42.01	71.68	46.65
Median	48.81	34.65	47.22	71.35	42.59
**Incidence**					
First week	22.50/1M	18.28/1M	491.49/1M	206.88/1M	191.27/1M
Last week	202.02/1M	38.75/1M	1053.90/1M	452.12/1M	251.30/1M
Mean	126.60/1M	23.22/1M	823.71/1M	449.05/1M	105.66/1M
Median	136.63/1M	21.10/1M	777.24/1M	504.25/1M	82.27/1M
**Deaths**					
First week	0.20/1M	0.99/1M	4,76/1M	3.07/1M	1.09/1M
Last week	1.23/1M	0.31/1M	11.23/1M	8.93/1M	1.22/1M
Mean	0.59/1M	0.56/1M	11.42/1M	5.71/1M	0.72/1M
Median	0.37/1M	0.53/1M	10.49/1M	4.91/1M	0.68/1M
**Vaccinations**					
First week	58.16/1H	36.40/1H[a]	6.94/1H[c]	62.05/1H	86.41/1H[f]
Last week	65.65/1H	40.61/1H[a]	-[d]	70.31/1H	89.48/1H[f]
Mean	62.22/1H	38.22/1H[b]	19.31/1[e]	65.27/1H	88.26/1H[g]
Median	62.23/1H	38.39/1H[b]	21.42/1H[e]	64.63/1H	88.48/1H[g]

Note. All data are based on COVID-19 dataset provided by Our World in Data (https://github.com/owid/covid-19-data/tree/master/public/data, accessed on 22 November 2022). Stringency index: a composite measure of nine response metrics that records the strictness of government measures (from 0 to 100; i.e., 100 = strictest response). Incidences: new cases smoothed per million. Deaths: new deaths smoothed per million. Vaccinations: people vaccinated per hundred. Due to missing data, some calculations regarding vaccination were based on smaller subsamples, namely: [a] *n* = 6. [b] *n* = 30. [c] *n* = 2. [d] people vaccinated per hundred not reported for last week of assessment: value 27.08 six days bevor end of data collection. [e] *n* = 59. [f] *n* = 1. [g] *n* = 12.

S1 Appendix. Detailed information on the methodology and participants' characteristics.

Independant variables

S2 Table. Overview of independent variables and their operationalisation.

Independent variable	Operationalisation
**Health-related characteristics**	
Health status	"How would you describe your current health?" (1 = *Very good*, 5 = *Very bad*)
COVID-19 infection	"Have you been infected (i.e., tested positive) with the coronavirus?" (0 = *No*, 1 = *Yes*)
Risk for severe course of COVID-19	"Do you think that you are at risk for severe or life-threatening symptoms of the coronavirus disease?" (0 = *No*, 1 = *Yes*)
History of mental health problems	"Have you ever been diagnosed with a mental disorder, e.g., depressive disorder or anxiety disorder?" (0 = *No*, 1 = *Yes, but I have recovered*, 2 = *Yes, I am currently affected*)
**Social factors**	
Face-to-face contact to loved ones	"How often do you have physical face-to-face contact to loved ones or friends?" (0 = *I have no face-to-face contact with other people*, 1 = *Less than once a week*, 2 = *Once a week,* 3 = *1-2 times a week*, 4 = *More than 3 times a week*)
Virtual contact to loved ones	"How often do you have virtual contact with loved ones or friends, e.g., by phone, Skype or Zoom?" (0 = *I have no contact by phone, skype etc*, 1 = *Less than once a week*, 2 = *Once a week,* 3 = *1-2 times a week*, 4 = *3-6 times a week*, 5 = *Everyday*)
Time spent at home	"Do you have spent more time at home due to the coronavirus pandemic?" (0 = *no*, 1 = *Yes, I have spent more time at home as precautionary measure [social distancing]*, 2 = *Yes, I have stayed at home in self-isolation because I have been infected myself,* 3 = *Yes, I have stayed at home because I had contact with infected people or have been in risk areas [quarantine], 4 = Not applicable)*
**Financial situation**	
Pandemic-related income loss	"Has the coronavirus pandemic reduced your monthly household income?" (0 = *No*, 1 = *Yes*)
Financial support	"Are you receiving financial support from the government to reduce the financial burden of the corona crisis?" (0 = *No*, 1 = *Yes*)

S1 Appendix. Detailed information on the methodology and participants' characteristics.

Inter- and intrarater agreement

S3 Table. Intra- and interrater agreement across countries.

	Austria	Croatia	Georgia	Greece	Portugal
	%	%	%	%	%
Interrater agreement					
T1	96.19	92.94	95.90	99.91	94.61
T2	97.35	96.64	95.78	99.79	95.56
Intrarater agreement					
T1	97.23	91.23	98.20	92.79	95.39
T2	93.68	95.03	97.72	95.10	95.02

S1 Appendix. Detailed information on the methodology and participants' characteristics.

Participants characteristics per time point

S4 Table. Health-related characteristics of the participants by country.

	T1					T2				
	AUT	CRO	GEO	GR	PT	AUT	CRO	GEO	GR	PT
	n (%)	*n* (%)	*n* (%)	*n* (%)	*n* (%)	*n* (%)	*n* (%)	*n* (%)	*n* (%)	*n* (%)
**Current health**										
Very good	149 (44.7)	125 (30.2)	23 (20.4)	68 (55.7)	12 (13.6)	121 (36.3)	102 (24.6)	20 (17.7)	55 (45.1)	11 (12.5)
Good	118 (35.4)	202 (48.8)	40 (35.4)	38 (31.1)	41 (46.6)	134 (40.2)	198 (47.8)	52 (46.0)	46 (37.7)	38 (43.2)
Satisfactory	54 (16.2)	76 (18.4)	40 (35.4)	15 (12.3)	34 (38.6)	61 (18.3)	98 (23.7)	31 (27.4)	16 (13.1)	35 (39.8)
Bad	11 (3.3)	10 (2.4)	9 (8.0)	1 (.8)	1 (1.1)	14 (4.2)	15 (3.6)	9 (8.0)	4 (3.3)	4 (4.5)
Very bad	1 (.3)	1 (.2)	1 (.9)	0 (0)	0 (0)	3 (.9)	1 (.2)	1 (.9)	1 (.8)	0 (0)
**Diagnosis of mental disorder**										
No	260 (78.1)	371 (89.6)	94 (83.2)	111 (91.0)	58 (65.9)	258 (77.5)	366 (88.4)	95 (84.1)	109 (89.3)	55 (62.5)
Yes, but recovered	52 (15.6)	29 (7.0)	8 (7.1)	6 (4.9)	16 (18.2)	54 (16.2)	25 (6.0)	12 (10.6)	7 (5.7)	20 (22.7)
Yes, currently affected	21 (6.3)	14 (3.4)	11 (9.7)	5 (4.1)	14 (15.9)	21 (6.3)	23 (5.6)	6 (5.3)	6 (4.9)	13 (14.8)
**Infected with COVID-19**										
No	330 (99.1)	413 (99.8)	113 (100)	121 (99.2)	87 (98.9)	306 (91.9)	313 (75.6)	83 (73.5)	112 (91.8)	80 (90.9)
Yes	3 (.9)	1 (.2)	0 (0)	1 (.8)	1 (1.1)	27 (8.1)	101 (24.4)	30 (26.5)	10 (8.2)	8 (9.1)
**At risk for severe COVID-19**										
No	270 (81.1)	323 (78.0)	92 (81.4)	96 (78.7)	76 (86.4)	289 (86.8)	341 (82.4)	98 (86.7)	103 (84.4)	82 (93.2)
Yes	63 (18.9)	91 (22.0)	21 (18.6)	26 (21.3)	12 (13.6)	44 (13.2)	73 (17.6)	15 (13.3)	19 (15.6)	6 (6.8)

Note. The number of participants per country corresponds to the total subsample size, as reported in Table 1 (AUT: *n* = 333; CRO: *n* = 414; GEO: *n* = 113; GR: *n* = 122; PT: *n* = 88).

S1 Appendix. Detailed information on the methodology and participants' characteristics.

S5 Table. Social environment of the participants by country.

	T1					T2				
	AUT	CRO	GEO	GR	PT	AUT	CRO	GEO	GR	PT
	n (%)	*n* (%)	*n* (%)	*n* (%)	*n* (%)	*n* (%)	*n* (%)	*n* (%)	*n* (%)	*n* (%)
**Face-to-face contact**										
No face-to-face contact	3 (.9)	54 (13.0)	34 (30.1)	5 (4.1)	29 (33.0)	2 (.6)	10 (2.4)	7 (6.2)	3 (2.5)	3 (3.4)
Less than once a week	52 (15.6)	157 (37.9)	40 (35.4)	29 (23.8)	34 (38.6)	52 (15.6)	83 (20.0)	45 (39.8)	17 (13.9)	15 (17.0)
Once a week	45 (13.5)	78 (18.8)	14 12.4)	17 (13.9)	10 (11.4)	44 (13.2)	75 (18.1)	14 (12.4)	17 (13.9)	24 (27.3)
1-2 times a week	112 (33.6)	56 (13.5)	18 (15.9)	31 (25.4)	10 (11.4)	124 (37.2)	112 (27.1)	31 (27.4)	36 (29.5)	22 (25.0)
More than 3 times a week	121 (36.3)	69 (16.7)	7 (6.2)	40 (32.8)	5 (5.7)	111 (33.3)	134 (32.4)	16 (14.2)	49 (40.2)	24 (27.3)
**Virtual contact**										
No virtual contact	4 (1.2)	9 (2.2)	1 (.9)	2 (1.6)	0 (0)	7 (2.1)	27 (6.5)	0 (0)	3 (2.5)	0 (0)
Less than once a week	35 (10.5)	23 (5.6)	6 (5.3)	12 (9.8)	0 (0)	38 (11.4)	44 (10.6)	5 (4.4)	17 (13.9)	7 (8.0)
Once a week	19 (5.7)	38 (9.2)	1 (.9)	7 (5.7)	2 (2.3)	41 (12.3)	55 (13.3)	8 (7.1)	7 (5.7)	5 (5.7)
1-2 times a week	59 (17.7)	48 (11.6)	12 (10.6)	28 (23.0)	10 (11.4)	62 (18.6)	78 (18.8)	10 (8.8)	30 (24.6)	13 (14.8)
3-6 times a week	104 (31.2)	84 (20.3)	13 (11.5)	23 (18.9)	15 (17.0)	88 (26.4)	72 (17.4)	23 (20.4)	23 (18.9)	17 (19.3)
Everyday	112 (33.6)	212 (51.2)	80 (70.8)	50 (41.0)	61 (69.3)	97 (29.1)	138 (33.3)	67 (59.3)	42 (34.4)	46 (52.3)
**More time spent at home**										
No	59 (17.7)	43 (10.4)	9 (8.0)	0 (0)	6 (6.8)	143 (42.9)	108 (26.1)	24 (21.2)	0 (0)	37 (42.0)
Yes (precaution)	265 (79.6)	339 (81.9)	102 (90.3)	15 (12.3)	72 (81.8)	163 (48.9)	189 (45.7)	80 (70.8)	65 (53.3)	43 (48.9)
Yes [a] (self-isolation)	0 (0)	3 (.7)	0 (0)	96 (78.7)	2 (2.3)	2 (.6)	53 (12.8)	3 (2.7)	51 (41.8)	0 (0)
Yes [b] (quarantine)	9 (2.7)	9 (2.2)	0 (0)	2 (1.6)	3 (3.4)	1 (.3)	32 (7.7)	0 (0)	3 (2.5)	2 (2.3)
Not applicable	0 (0)	20 (4.8)	2 (1.8)	9 (7.4)	5 (5.7)	24 (7.2)	32 (7.7)	6 (5.3)	3 (2.5)	6 (6.8)

[a] Due to infection. [b] Due to contact with infected people or for being in risk areas.

S1 Appendix. Detailed information on the methodology and participants' characteristics.

S6 Table. Pandemic-related financial situation of participants by country.

	**T1**					**T2**				
	AUT	CRO	GEO	GR	PT	AUT	CRO	GEO	GR	PT
	n (%)	*n* (%)	*n* (%)	*n* (%)	*n* (%)	*n* (%)	*n* (%)	*n* (%)	*n* (%)	*n* (%)
**Reduced monthly household income**										
Yes	77 (23.1)	166 (40.1)	57 (50.4)	48 (39.3)	22 (25.0)	53 (15.9)	92 (22.2)	46 (40.7)	45 (36.9)	8 (9.1)
No	256 (76.9)	248 (59.9)	56 (49.6)	74 (60.7)	66 (75.0)	280 (84.1)	322 (77.8)	67 (59.3)	77 (63.1)	80 (90.9)
**Financial support**										
Yes	13 (16.9)	9 (5.4)	6 (10.5)	13 (27.1)	4 (18.2)	10 (18.9)	4 (4.3)	2 (4.3)	6 (13.3)	0 (0)
No	64 (83.1)	157 (94.6)	51 (89.5)	35 (72.9)	18 (81.8)	43 (81.1)	88 (95.7)	44 (95.7)	39 (86.7)	8 (100)

Note. The number of participants per country corresponds to the total subsample size, as reported in Table 1 (AUT: *n* = 333; CRO: *n* = 414; GEO: *n* = 113; GR: *n* = 122; PT: *n* = 88).
