# Supplementary material for: Shaped by the COVID-19 pandemic: Psychological responses from a subjective perspective–A longitudinal mixed-methods study across five European countries
Source: PLoS One. 2023 Apr 25;18(4):e0285078. doi: 10.1371/journal.pone.0285078 (PMC10128933; doi:10.1371/journal.pone.0285078)
Supplement: S2 Appendix — (PDF) [file pone.0285078.s002.pdf]

S2 Appendix. Themes and categories (T1 and T2).

Note: The parts indicated in red represent changes made in T2 (e.g. newly emerged categories, changes in wording, additional explanations)

List of themes

The most stressful event

Themes	Categories
**1. Restrictions and changes in daily life**	Shutdown and circumstances in general<br>Disturbed daily structure and the need to adapt<br>Social restrictions<br>Having to stay at home/Stay at home measures<br>Restricted leisure activities<br>Travel restrictions<br>Restrictions on religious activity<br>Face masks and other COVID-19 measures
**2. COVID-19 and other health issues**	COVID-19 and related deaths and illnesses<br>Health problems
**3. Vaccination issues**	Vaccination issues
**4. Emotional distress**	Emotional burden<br>Uncertainty/insecurity
**5. Work and finances**	Work-related burden<br>Economic problems
**6. Burden related to loved ones**	Home-schooling and distance learning<br>Reconciling childcare/home-schooling with working from home<br>Burden/stressors related to loved ones
**7. Societal impact**	Reactions of the population<br>Violation of human rights<br>Stigmatisation due to COVID-19
**8. Pandemic management and communication**	Incompetent pandemic management by institutions<br>Insufficient health care<br>Inadequate information<br>Inadequate media handling
**9. Other burden**	
**10. No stressful events**	
**11. Not specified**	

S2 Appendix. Themes and categories (T1 and T2).

Negative aspects of the pandemic

Themes	Categories
**1. Restrictions and changes in daily life**	Shutdown and circumstances in general<br>Disturbed daily structure and the need to adapt<br>Social restrictions<br>Having to stay at home/Stay at home measures<br>Restricted leisure activities<br>Travel restrictions<br>Restrictions on religious activity<br>Face masks and other COVID-19 measures
**2. COVID-19 and other health issues**	COVID-19 and related deaths and illnesses<br>Health problems
**3. Vaccination issues**	Vaccination issues
**4. Emotional distress**	Emotional burden<br>Uncertainty/insecurity<br>Endlessness
**5. Work and finances**	Work-related burden<br>Economic problems
**6. Burden related to loved ones**	Home-schooling and distance learning<br>Reconciling childcare/home-schooling with working from home<br>Burden/stressors related to loved ones
**7. Societal impact**	Reactions of the population<br>Violation of human rights<br>Stigmatisation due to COVID-19<br>Impact on countries with weak social systems<br>Impact on specific groups<br>Division of the society
**8. Pandemic management and communication**	Incompetent pandemic management by institutions<br>Insufficient health care<br>Inadequate information<br>Inadequate media handling<br>Politicisation of the pandemic<br>Loss of trust in science/healthcare
**9. Other negative aspects**	
**10. No negative aspects**	
**11. Not specified**	

S2 Appendix. Themes and categories (T1 and T2).

Positive aspects of the pandemic

Themes	Categories
**1. Reflection and growth**	Individual and community growth<br>Self-awareness<br>Rethinking<br>Appreciation/gratitude<br>Risk/danger awareness<br>Awareness for better hygiene<br>Focussing on the essentials<br>Taking care of yourself
**2. Opportunity for meaningful/enjoyable activities**	Meaningful/enjoyable daily activities<br>Having more time<br>More peace in life
**3. Environmental effects**	Less travel, traffic and tourism<br>Impact on climate/nature
**4. Benefits on interpersonal level**	Social cohesion<br>Family quality time/bonding<br>Benefits of social constraints
**5. Digitalisation and working/studying from home**	Digitalisation<br>Home-office/Distance learning
**6. Competent pandemic management**	Competent pandemic management of institutions
**7. Vaccination/Access to vaccination**	Vaccination/Access to vaccination
**8. Other positive aspects**	
**9. No positive aspects**	
**10. Not specified**	

S2 Appendix. Themes and categories (T1 and T2).

Recommendations for dealing with the pandemic

Themes	Categories
**1. Beneficial behavioural adjustment**	Use your mind<br>Inform adequately<br>Look after yourself<br>Look after your health<br>Stay active/balance<br>Regular daily routine<br>New habits<br>Setting goals<br>Adherence to containment measures<br>Being locally rooted
**2. Beneficial cognitive-emotional strategies**	Positive inner attitude (Calmness, Optimism, Acceptance)<br>Religion and spirituality<br>Modesty and simplicity
**3. Social support**	Keeping contact with others<br>Mutual help/support
**4. Political recommendations**	Political recommendations
**5. Get vaccinated**	Get vaccinated
**6. Other recommendations**	
**7. No recommendations**	
**8. Not specified**	

S2 Appendix. Themes and categories (T1 and T2).

List of categories

The most stressful event

**Shutdown and circumstances in general**	This category includes all statements in which shutdown/lockdown per se and the associated restriction of personal freedom were named as the most stressful event. Also included here are statements that refer to COVID-19 related circumstances in general, rather than to a specific event.<br><br>**Anchor examples**: "There is no one event. More like the circumstance all together...", "Shutdown."
**Violation of human rights**	This category covers a wide range of statements about violation of human rights, excessive use of force by law and police enforcement, misuse of power, and too strict policing that result in human rights issues.<br><br>**Anchor examples:** „police enforcement through undemocratic measures“, „dictatorship“, „being treated like slaves“.
**Social restrictions**	This category includes a wide range of stressors with regard to social constraints. These are all stressors that have arisen due to the limitations of social contact within the COVID-19 protective measures. These include, but are not limited to, social restrictions in general, isolation, reduced/no visitation opportunities, lack of physical contact, inability to say goodbye to a deceased person appropriately.<br><br>**Anchor examples**: "Not being able to meet friends," "Strict reduction of social contact," "No visits from own children and grandchildren," "Impossibility of proper farewell/funerals," "Not being allowed to hug my mother."
**Having to stay at home/ Stay at home measures**	This category includes responses from those for whom having to stay at home was the most stressful. Also meant are stressors related to lack of space or lack of a place to retreat („safe haven“).<br><br>**Anchor examples**: "confined within my own 4 walls," "not having a patio or balcony."
**Restricted leisure activities**	Statements that refer to limited leisure opportunities are subsumed here. "Leisure activities" refers to sports, club and cultural activities as well as outdoor activities. Not meant are activities related to meeting the necessary basic needs of daily life (e.g., shopping at supermarkets/pharmacies, visits to the doctor). Shopping is also understood as a leisure activity and is therefore to be distinguished from necessary shopping.<br>Differentiation from social constraints: Leisure activity must be paramount, while the social aspect is not mentioned. Accordingly, "closing of the pensioners' club" is coded as a restricted leisure activity, while "no meeting with senior citizens group" is coded as a social constraint, since the social aspect is paramount in the latter.

S2 Appendix. Themes and categories (T1 and T2).

	**Anchor examples**: "The park near my apartment was closed, which was bad for me," "cancellation of cultural events," "limited leisure activities," "no shopping"
**Travel restrictions**	Travel restrictions (domestic and international) per se, as well as none/restricted opportunities to visit loved ones due to travel restrictions, are recorded here. Cancellations of travel and inability to travel are also included, with home travel included here.<br>**Anchor examples**: "Not being able to visit life partner abroad," "No travel," "Not being able to travel to family in another state."
**COVID-19 and related deaths and illnesses**	Answers regarding COVID-19 infections and deaths, including increases in infections and figures for corona virus, fall here. COVID-19 per se as well as associated health threats, long COVID, and reinfection are also included here.<br><br>**Anchor examples**: "high infection numbers", "the many deaths", "the daily information about the wave of infections", "symptoms of long covid"
**Vaccination issues**	This category includes statements about the vaccination and the population's reactions to the possibility to get vaccinated. It also includes statements referring to restrictions for those not vaccinated, the perceived or real pressure to get vaccinated and vaccine hesitancy.<br><br>**Anchor examples**: "friends, colleagues who refuse vaccination", "the decreasing willingness of many to be vaccinated", "being allowed to do certain things only if you are vaccinated", "vaccination refusers"
**Emotional burden**	In this category, all kinds of emotional burden that were explicitly mentioned are recorded. I.e. all feelings that were stressful for the participants (perceived/felt as a burden) fall into this category. Meant are, among others, fear, demotivation and feeling of loneliness.<br><br>**Anchor examples**: "demotivation by being at home", "fear of the invisible enemy".
**Uncertainty/insecurity**	When uncertainty and insecurity are mentioned directly or indirectly as the most stressful events, they are subsumed here. Both uncertainty/uncertainty in general and in relation to specific areas of life/situations are meant here.<br><br>**Anchor examples**: "uncertainty about the future", "for a long time it was not clear whether I would be allowed to attend the funeral".
**Work-related burden**	This category contains any work-related burden. All events that happened in the work context fall in here.<br><br>**Anchor examples**: "contact with covid patients," "the transition from work to home office," "I was fired for 1 month."
**Economic problems**	This category contains statements about economic problems related to the Covid-19 pandemic. This includes responses related to a) overall economic impact, b) economic impact on specific sectors (e.g., firms), c) job loss/unemployment.

S2 Appendix. Themes and categories (T1 and T2).

	**Anchor examples**: "that the economy suffered severe downturns," "that many people lost their jobs."
**Home-schooling and distance learning**	This category includes all statements regarding the impact of the pandemic on education.<br>Closure of schools/university as well as negative evaluations of learning from home are included here.<br><br>**Anchor examples:** „challenging online exams at the university“, „children are not allowed to go to school“, „chaotic school situation“
**Reconciling childcare/ home-schooling with working from home**	This category signals the difficulty in reconciling working from home or housework with childcare or helping children attend home school or distance learning. This category shows how complex it is to adapt to and balance these two realities that are experienced simultaneously.<br><br>**Anchor examples:** „Managing time between household duties, helping my children during classes and activities that were proposed to them (that usually lasted all day“, „Staying home with my 4-year-old son for two and a half months while working from home“, „Reconciling teleworking with taking care of my 5 year old daughter“
**Burden/stressors related to loved ones**	This category includes all stressors in the circle of close people (family, partners, relatives, roommates, friends). These range from conflicts to illnesses and deaths that are not directly COVID-19 related.<br><br>**Anchor examples**: "partner away a lot," "husband in hospital with real flu," "increased arguments with my boyfriend," "death of grandma."
**Health problems**	This category contains all health-related problems of the participants that are not directly related to COVID-19.<br><br>**Anchor examples:** „staying at hospital“, „fluctuating blood pressure“
**Reactions of the population**	Here, stressors are subsumed that result from reactions of the population. This refers to those groups of people who, from the participants point of view, behave inappropriately/show inappropriate reactions (or have changed for the worse). These could be, for example, people who do not comply with measures or panic.<br><br>**Anchor examples**: "the grocery stores were emptied (panic hoarding)" "unnecessary discussions with anti-maskers," "other people not showing consideration."
**Stigmatisation due to COVID-19**	This category includes all statements which imply that people who were infected with Covid-19 were in any way mistreated or stigmatized. It also includes stigmatization of people who could potentially be infected, e.g. due to being in contact with Covid-19 patients or travelling abroad.<br><br>**Anchor examples**: „Stigmatisation of people who were infected, or were in contact with the infected, or even travelled abroad…“

S2 Appendix. Themes and categories (T1 and T2).

**Incompetent pandemic management by institutions**	This category includes statements about what participants perceive to be incompetent institutional pandemic management. Institutions are here defined as government as well as authorities and educational institutions.<br><br>**Anchor examples**: "The fact that there were no legally binding regulations", "Stupid reports from the opposition", "The disorganization of the universities".
**Insufficient health care**	This category includes all statements referring to insufficient health care being provided to participants, or some parts of the health care system being affected negatively by the pandemic. These statements could be general or specific.<br><br>**Anchor examples**: „Poor access to health care“, „Inability to conduct certain health examinations“, „Closing of hospitals“, „Disfunction of the healthcare system during the pandemic“.
**Information**	This category includes all statements that refer to a lack of information, too much information or contradictory information about the pandemic without explicit mention of the media or other institutions. These statements could be general or specific.<br><br>**Anchor examples**: „Insufficient information on causes, symptoms, disease progression, treatment/prevention…“, „not enough information“, „contradictory information“, „lack of information on the disease“.
**Media handling**	This category includes statements about, from the participants' point of view, inadequate handling of pandemic-related issues in the media. Media are understood as technical communication channels incl. print media, audio-visual media as well as online media.<br><br>**Anchor examples**: "Bullshit messages from media".
**Disturbed daily structure and the need to adapt**	This category includes all statements regarding the disturbances of daily structure, daily routines or lifestyle due to pandemics. It also includes statements related to the need to adapt to a new lifestyle.<br><br>**Anchor examples**: „Loss of the daily structure“, „Changes in usual everyday rhythm“, „Lack of life structure“, „Disturbed time structure of daily activities“, „The need to adapt to a new situation“, „Hard to „catch“ a new rhythm“.
**Face masks and other COVID-19 measures**	All answers related to face masks, testing, physical distancing, and similar protective measures are included in this category.<br><br>**Anchor examples**: „wearing face masks“,“covid testing“
**Restrictions on religious activity**	This category includes specific form of stressor with regard to restriction of religious activity. It includes the inability to participate in religious ceremonies and activities, such as attending mass, meetings of a religious group, etc.<br>**Anchor examples:** "No possibility of going to church", "No Holy Thursday mass and no Good Friday services"
**Other burden**	Stressful events that cannot be assigned to any other category are subsumed here. These are mostly very individual events that are highly unlikely to have occurred to other participants or

S2 Appendix. Themes and categories (T1 and T2).

	would probably not be described as particularly stressful by other participants.<br><br>Events that were mentioned more than once are not subsumed here. If necessary, a separate category should be formed.
**No stressful events**	If participants indicated that they had not experienced a stressful event, their responses are subsumed here.<br><br>**Anchor examples**: "I have not had a stressful event because I can always keep myself busy on my own", "Did not have any stress".
**Not specified**	If nothing was specified, this category is chosen.<br>Both "not specified", "n/a", "n/a" and any special characters (e.g. "/", "...") count as an answer for this category.

S2 Appendix. Themes and categories (T1 and T2).

Negative aspects of the pandemic

**Shutdown and circumstances in general**	This category includes all statements in which shutdown/lockdown per se and the associated restriction of personal freedom were named as the most stressful event. Also included here are statements that refer to COVID-19 related circumstances in general, rather than to a specific event.<br><br>**Anchor examples**: „being restricted“, „Lockdown“
**Violation of human rights**	This category covers a wide range of statements about violation of human rights, excessive use of force by law and police enforcement, misuse of power, and too strict policing that result in human rights issues.<br><br>**Anchor examples:** „police enforcement through undemocratic measures“, „dictatorship“, „being treated like slaves“.
**Social restrictions**	This category includes a wide range of stressors with regard to social constraints. These are all stressors that have arisen due to the limitations of social contact within the COVID-19 protective measures. These include, but are not limited to, social restrictions in general, isolation, reduced/no visitation opportunities, lack of physical contact, inability to say goodbye appropriately, missing friends/family.<br><br>**Anchor examples***:* "the prohibition of a warm greeting with grandparents," "harder to meet new people“
**Having to stay at home/ Stay at home measures**	This category includes responses from those for whom having to stay at home was the most stressful. Also meant are stressors related to lack of space or lack of a place to retreat („safe haven“).<br><br>**Anchor examples**: "confined within my own 4 walls," "not having a patio or balcony."
**Restricted leisure activities**	Answers related to limited leisure activities are subsumed here. "Leisure activities" refers to sports, club and cultural activities as well as outdoor activities. Not meant are activities related to meeting the necessary basic needs of daily life (e.g., shopping at supermarkets/pharmacies, visits to the doctor). Shopping is also understood as a leisure activity and is to be distinguished from necessary purchases.<br><br>**Anchor examples**: "the cultural desert," "fewer leisure activities such as movies..."
**Travel restrictions**	Travel restrictions (domestic and international) per se, as well as none/restricted opportunities to visit loved ones due to travel restrictions, are recorded here. Cancellations of travel and inability to travel are also included, with home travel included here.<br><br>**Anchor examples**: "no vacations", "travel warnings - hardly any opportunities to meet friends and family abroad".
**COVID-19 and related deaths and illnesses**	Answers regarding COVID-19 infections and deaths, including increases in infections and figures for corona virus, fall here.

S2 Appendix. Themes and categories (T1 and T2).

	COVID-19 per se as well as associated health threats, long COVID, and reinfection are also included here.<br><br>**Anchor examples**: "high infection numbers", "the many deaths", "the daily information about the wave of infections", "symptoms of long covid"
**Vaccination issues**	This category includes statements about the vaccination and the population’s reactions to the possibility to get vaccinated. It also includes statements referring to restrictions for those not vaccinated, the perceived or real pressure to get vaccinated and vaccine hesitancy.<br><br>**Anchor examples***:* "friends, colleagues who refuse vaccination", "the decreasing willingness of many to be vaccinated", "being allowed to do certain things only if you are vaccinated", "vaccination refusers"
**Emotional burden**	This category includes all kinds of emotional burden that were explicitly mentioned. In other words, all feelings that were stressful (perceived/felt as a burden) for the respondents are included here. These include fear and feelings of loneliness.<br>It is irrelevant whether the negative feelings are the respondent's own or those of other persons.<br><br>**Anchor examples**: "that many people have to worry about their existence", "stress", "partly fear for at-risk groups", "loneliness".
**Uncertainty/insecurity**	When uncertainty and insecurity are mentioned directly or indirectly as the most stressful events, they are subsumed here. Both uncertainty/uncertainty in general and in relation to specific areas of life/situations are meant here.<br><br>**Anchor examples**: "general uncertainty - what will happen next?", "hard to plan ahead", "who will get infected, how will it turn out?"
**Endlessness**	This category includes statements concerning the (unexpected) length of the pandemic (i.e., never-ending story). Specific statements that address the unpredictable end of the pandemic are also subsumed here.<br><br>**Anchor examples***:* "No end in sight", "The end that cannot be estimated"
**Work-related burden**	This category contains any work-related burden/problem. All events that happened in the work context fall in here.<br><br>**Anchor examples:** „elevated stress at work“, „I have worked more than ever in my life“, „working with face mask“
**Economic problems**	This category contains statements about economic problems related to the Covid-19 pandemic. This includes responses related to a) overall economic impact, b) economic impact on specific sectors (e.g., firms), c) job loss/unemployment.<br><br>**Anchor examples**: "that the economy suffered severe downturns," "that many people lost their jobs."

S2 Appendix. Themes and categories (T1 and T2).

**Home-schooling and distance learning**	This category includes all statements regarding the impact of the pandemic on education.<br>Closure of schools/university as well as negative evaluations of learning from home are included here.<br><br>**Anchor examples:** „challenging online exams at the university“, „children are not allowed to go to school“, „chaotic school situation“
**Reconciling childcare/ home-schooling with working from home**	This category signals the difficulty in reconciling working from home or housework with childcare or helping children attend home-school or distance learning. This category shows how complex it is to adapt to and balance these two realities that are experienced simultaneously.<br><br>**Anchor examples:** „Managing time between household duties, helping my children during classes and activities that were proposed to them (that usually lasted all day“, „Staying home with my 4-year-old son for two and a half months while working from home“, „Reconciling teleworking with taking care of my 5 year old daughter“
**Burden/stressors related to loved ones**	This category includes all stressors in the circle of close people (family, partners, relatives, roommates, friends). These range from conflicts to illnesses and deaths that are not directly COVID-19 related.<br><br>**Anchor examples**: "partner away a lot," "husband in hospital with real flu," "increased arguments with my boyfriend," "death of grandma."
**Impact on countries with weak social systems**	This category contains statements about the impact of the pandemic on countries with weak social systems (often referred to as developing countries). The statements can refer to specific aspects (e.g., lack of medical care), but also more generally to unequal opportunities in dealing with COVID-19.<br><br>**Anchor examples**: "the impact on countries without a social system, such as India, China, etc., where people die of malnutrition and hunger instead of the virus", "poor medical care and poor government management in developing countries."
**Impact on specific target groups**	This category includes all statements regarding the impact of the pandemic on specific target groups, such as: women, elderly, refugees etc.<br><br>**Anchor examples:** „bad for young people“, „hard for old people and their relatives“, „exploitation relationships will remain such and women in particular will again be the bigger losers than men“
**Health problems**	This category contains all health-related problems of the participants that are not directly related to COVID-19.<br><br>**Anchor examples:** „staying at hospital“, „fluctuating blood pressure“

S2 Appendix. Themes and categories (T1 and T2).

**Reactions of the population**	Here, stressors are subsumed that result from reactions of the population. This refers to those groups of people who, from the participants point of view, behave inappropriately/show inappropriate reactions (or have changed for the worse). These could be, for example, people who do not comply with measures or panic.<br><br>**Anchor examples**: "That it is unfortunately not taken seriously enough by many", "that many people fall seriously ill or even have to die from it due to the unreasonableness of others", "division of society, denunciators, loss of empathy and cohesion".
**Division of the society**	This category includes statements about the division of society into two camps. This division can be understood from a political perspective as well as concerning a person's views around the corona virus or his/her vaccine status.<br><br>**Anchor examples***:* "the division of the society ", "Polarisation of the society into 'corona deniers' and 'coronaphobes' ", "division of the society by dividing it into vaccination opponents and proponents."
**Stigmatisation due to COVID-19**	This category includes all statements which imply that people who were infected with Covid-19 were in any way mistreated or stigmatized. It also includes stigmatization of people who could potentially be infected, e.g. due to being in contact with Covid-19 patients or travelling abroad.<br><br>**Anchor examples**: „Stigmatisation of people who were infected, or were in contact with the infected, or even travelled abroad…“
**Incompetent pandemic management by institutions**	This category includes statements about what participants perceive to be incompetent institutional pandemic management. Institutions are here defined as government as well as authorities and educational institutions.<br><br>**Anchor examples**: "other countries' failure to act or acting too late," "the government's muppet show," "populist chancellor."
**Politicisation of the pandemic**	This category includes responses indicating concerns that politicians and political parties are using the pandemic or containment measures to achieve their own goals or score political points. It also includes statements regarding the excessive influence of politics to containment and health measures.<br><br>**Anchor examples:** „politicisation of the measures, i.e. an attempt to bring into power ideological measures using health as an excuse“; „using the pandemics in political purposes“, „the influence of politics on epidemiological measures“.
**Loss of trust in science/healthcare**	Statements not directly related to incompetent managing of the pandemic by the institutions, but about specific loss of trust into healthcare and science that they can offer solutions which will contribute to ending the crisis; i.e. loss of trust into their integrity.

S2 Appendix. Themes and categories (T1 and T2).

	**Anchor examples:** „Listing all people who have died as if they died of Covid“, „lower trust in science“, “mistrust in scientists and scientific solutions for coronavirus pandemic”
**Insufficient health care**	This category includes all statements referring to insufficient health care being provided to participants, or some parts of the health care system being affected negatively by the pandemic. These statements could be general or specific.<br><br>**Anchor examples**: „Poor access to health care“, „Inability to conduct certain health examinations“, „Closing of hospitals“, „Disfunction of the healthcare system during the pandemic“.
**Information**	This category includes all statements that refer to a lack of information, too much information or contradictory information about the pandemic without explicit mention of the media or other institutions. These statements could be general or specific.<br><br>**Anchor examples**: „Insufficient information on causes, symptoms, disease progression, treatment/prevention…“, „not enough information“, „contradictory information“, „lack of information on the disease“.
**Media handling**	This category includes statements about, from the participants' point of view, inadequate handling of pandemic-related issues in the media. Media are understood as technical communication channels incl. print media, audio-visual media as well as online media.<br><br>**Anchor examples**: "scaremongering in the media", "the many different pieces of information in all media"
**Disturbed daily structure and the need to adapt**	This category includes all statements regarding the disturbances of daily structure, daily routines or lifestyle due to pandemics. It also includes statements related to the need to adapt to a new lifestyle.<br><br>**Anchor examples**: „Loss of the daily structure“, „Changes in usual everyday rhythm“, „Lack of life structure“, „Disturbed time structure of daily activities“, „The need to adapt to a new situation“, „Hard to „catch“ a new rhythm“.
**Face masks and other COVID-19 measures**	All answers related to face masks, testing, physical distancing, and similar protective measures are included in this category.<br><br>**Anchor examples**: „wearing face masks“,“covid testing“
**Restrictions on religious activity**	This category includes specific form of stressor with regard to restriction of religious activity. It includes the inability to participate in religious ceremonies and activities, such as attending mass, meetings of a religious group, etc.<br><br>**Anchor examples:** "No possibility of going to church", "No Holy Thursday mass and no Good Friday services"
**Other negative aspects**	Negative aspects that cannot be assigned to any other category are subsumed here. These are mostly very individual aspects that other participants are unlikely to find particularly negative about the pandemic.

S2 Appendix. Themes and categories (T1 and T2).

	Events that were mentioned more than once are not subsumed here. If necessary, a separate category should be formed.
**No negative aspects**	If participants indicated they found nothing negative about the pandemic, their responses are subsumed here.<br><br>**Anchor examples**: "nothing"
**Not specified**	If nothing was specified, this category is chosen. Both "not specified", "n/a", "n/a" and any special characters (e.g. "/", "...") count as an answer for this category.

S2 Appendix. Themes and categories (T1 and T2).

Positive aspects of the pandemic

**Individual and community growth**	This category describes learning experiences made in the course of the pandemic. It is about newly learned skills/attitudes and creative solutions. It also includes new concrete insights. It refers to one's own learning experiences as well as those of other people.<br><br>**Anchor examples**: "that I have learned to deal with situations that are outside my sphere of influence", "In addition, I find the creativity of many people, how they manage to come to income, very positive".
**Self-awareness**	This category includes all statements regarding positive evaluations of increased self-awareness or the process of introspection without explicit mention of the outcome of the process.<br><br>**Anchor examples**: „focus on internal processes", „„turning" towards oneself".
**Rethinking**	This category describes a restructuring of tasks, desires, or options according to their importance, on an individual or societal level.<br><br>**Anchor examples**: "noticing what is really important," "getting people to rethink," "rethinking priorities."
**Focusing on the essentials**	This category covers answers regarding modesty and to limiting oneself to essentials. Positive aspects specifically addressing to focus moderation and on what is essential/important to oneself are subsumed here.<br><br>**Anchor examples***:* "living minimalistic again due to restrictions and recognising what is important in life (especially health - both physical and mental)", "focusing on the essentials/important things"
**Appreciation/gratitude**	This category includes recognition and positive evaluations of what is available to the individual (e.g., health, home) or the society (e.g., health care system).<br><br>**Anchor examples**: "appreciation of health", "appreciation of the things and healthcare we have here, especially compared to countries that are still suffering and are much more suffering from the pandemic and its aftermath."
**Risk/danger awareness**	This category describes the development of an awareness of dangers associated with Covid-19. In addition, it includes the consequence of this increased awareness at the behavioural level. This danger may be current or future, individual or collective.<br><br>**Anchor examples**: "Contemplation that a danger could be imminent", "People become more cautious".
**Meaningful/enjoyable daily activities**	Answers related to meaningful and/or enjoyable activities are recorded here. These include, among others: reading, spending time in garden, cooking, doing sports.

S2 Appendix. Themes and categories (T1 and T2).

	*Whereas category „Having more time" focuses on *having more time resources*, the statements included here focus on *undertaking/actively engaging/doing pleasant activities*.<br><br>**Anchor examples:** „baking and exchanging recipes", „lot to read", „new cultivation of plants"
**Having more time**	Here, the experiences of having more time are subsumed. This includes having more time for oneself (in the sense of self-care) or others, and being able to spend more time at home (which in this case is experienced as positive).<br><br>**Anchor examples**: "More time to do things I like," "More time with partner and pet."
**More peace in life**	This category describes an everyday state characterized by less sensory overload, deceleration and less stress.<br><br>**Anchor examples**: "calming down" "life has become calmer."
**Less travel, traffic and tourism**	This category contains statements regarding the reduction of travels, traffic and tourism as a positive consequence of the pandemic.<br><br>**Anchor examples**: „reduction of pointless travels", „less traffic", „tourism is held back a little"
**Impact on climate/nature**	If effects on climate and/or nature are mentioned as positive aspects of the pandemic, they are subsumed here. In addition, statements that refer to more awareness of climate and environmental protection are recorded here.<br><br>**Anchor examples**: "Less environmental degradation," "The considerations regarding climate protection," "The partial recovery of nature."
**Better hygiene**	This category contains the statements regarding better hygiene conditions and better awareness of the importance of hygiene.<br><br>**Anchor examples:** „hopefully everyone learned to wash hands properly", „public transport and surfaces are being cleaned more often"
**Taking care of yourself**	Statements about the need for self-care, caring about own health or own hygiene*, which do not clearly express sense of gratitude for being healthy / for own wellbeing<br><br>**Anchor examples***:* "better caring for health", "self-care"<br><br>*Answers about better hygiene on a more general/societal level should be coded as "better hygiene".
**Social cohesion**	This category covers a wide range of prosocial behaviour within the various social groups (circle of friends, work group, society, humanity) in the wake of the pandemic. This is described as cohesion, empathy, thoughtfulness, and helpfulness.<br>*Please note:* Responses regarding the cohesion within families are subsumed in the category „family quality time/bonding"

S2 Appendix. Themes and categories (T1 and T2).

	**Anchor examples**: "solidarity in the society", "the helpfulness among people", "the worldwide cohesion and fight against the same opponent"
**Family quality time/bonding**	This category covers a wide range of statements about having or spending quality time with one's family. It also refers to the family bonding, family cohesion and families coming together in the course of the pandemic.<br><br>**Anchor examples:** „more quality time with my husband", „family bonding", „getting closer with family"
**Benefits of social constraints**	This category contains personally experienced positive aspects of social restrictions as one of the containment measures.<br><br>**Anchor examples**: "No social obligations," "Certain personal harassment cease (e.g., salesperson at the door)."
**Digitalization**	This category includes learning experiences using digital media as well as the benefits of online tools (e.g., global outreach)<br><br>**Anchor examples**: "I learned how to use Skype and ZOOM," "Many online offerings such as webinars connect/engage the whole world."
**Home Office/Distance Learning**	This category includes all positive evaluations of working/teaching/learning from home.<br><br>**Anchor examples**: "Work from home," "Home learning."
**Competent pandemic management of institutions**	This category includes positive evaluations of the government's pandemic management (including containment measures, financial packages) but also expert-lay communication on Covid-19 topics.<br><br>**Anchor examples**: "The reasonable behaviour of the Austrian government", "The factual information of individual expert virologists".
**Vaccination/Access to vaccination**	This category covers a wide range of statements around the vaccines development and the possibility to get vaccinated.<br><br>**Anchor examples***:* "The vaccination," "That hopefully the vaccinations will help and work against the virus.", "The willingness of many Austrians to get vaccinated."
**Other positive aspects**	Positive aspects that cannot be assigned to any other category are subsumed here. These are mostly very individual aspects that other participants are unlikely to find particularly positive about the pandemic.<br>Events that were mentioned more than once are not subsumed here. If necessary, a separate category should be formed.
**No positive aspects**	If participants indicated that they did not see any positive aspects, their responses are subsumed here.<br><br>**Anchor examples:** "There is nothing positive," "Absolutely nothing."
**Not specified**	If nothing was specified, this category is chosen.<br>Both "no indication", "n/a", "n/a" and any special characters (e.g. "/", "...") count as an answer for this category.

S2 Appendix. Themes and categories (T1 and T2).

S2 Appendix. Themes and categories (T1 and T2).

Recommendations for dealing with the pandemic

**Positive inner attitude**<br><br>***At T2, this category was divided into three subcategories for better comprehensibility:***	This category includes the recommendation of a resilience promoting attitude on how individuals can evaluate and respond to pandemic related events (in relation to their own lives and beyond). The recommended attitude is among others characterized by caution, staying calm, positive thinking, confidence, serenity, mindfulness, and gratitude.<br><br>**Anchor examples**: "Don't panic," "Instead of just thinking about limitations all the time, focus on things and activities that are possible."
**Calmness and patience** (subcategory of "positive inner attitude)	This category includes the recommendation promoting a calm and patient attitude in response to pandemic related events (regarding one's own life and beyond). The recommended attitude focuses on practicing patience, calmness, ease and caution.<br><br>**Anchor examples***:* "serenity and seriousness," "do not let yourself get crazy," "stay cool"
**Optimism** (subcategory of "positive inner attitude)	This category includes the recommendation promoting a positive and optimistic attitude in response to pandemic related events (with regard to one's own life and beyond). The recommended attitude focuses on openness, positivity, optimism, confidence/trust into future.<br><br>**Anchor examples***:* "Trust in good future, optimism", "Try to see the small positive things in everyday life", "Make the best of it".
**Acceptance** (subcategory of "positive inner attitude)	This category includes the recommendation to accept the situation the way it is (with regard to one's own life and beyond). The recommended attitude focuses on accepting the reality and make the best out of it, given we cannot change it.<br><br>**Anchor examples:** "accept it", "Make the best of it - you can't change it anyway"
**Use your mind**	This category describes the recommendation to make use of one's own mind/intelligence, e.g. also to steel oneself against conspiracy theories.<br><br>**Anchor examples**: "common sense and thinking would be a good thing", "be smart and don't be fooled by conspiracy theorists".
**Look after yourself**	This category covers the recommendations to perceive, respect and pursue one's own needs. In contrast to the category "Stay active/balance", this category does not include concrete examples of how to practice self-care, as this is to be individually tailored. Striving for good health is also coded separately (category "Look after your health").<br><br>**Anchor examples**: "Look after yourself, you don't have to optimize yourself in lockdown", "Take time for yourself".
**Inform adequately**	All recommendations regarding adequate informing about the pandemic are include into this category. These range from

S2 Appendix. Themes and categories (T1 and T2).

	general recommendations to stay well informed to more specific recommendations to consume only reliable sources of information. Recommendations to reduce media consumption are also recorded here.<br><br>**Anchor examples:** „obtain comprehensive information from various sources“, „don't spend too much time in front of TV“, „use mass media only very selectively“
**Stay active/balance**	Here, recommendations are subsumed that involve actively shaping everyday life, also in terms of feel-good activities, pursuing one's own interests and (meaningful) activities: Household, hobbies, sports/exercise, relaxation techniques, going out (into nature).<br><br>**Anchor examples**: "Go out a lot, into the fresh air alone, take in sunshine," "Yoga/meditation."
**Look after your health**	Recommendations for maintaining health and strengthening the immune system are included into this category. This can be made as a general recommendation or as a specific recommendation (e.g., eat healthy food). Specific recommendations that fall under other categories (e.g., staying active) are coded there.<br><br>**Anchor examples**: "Try to take care of your health and immune system autonomously," "Have a healthy diet."
**Regular daily routine**	This category contains recommendations that suggest a regular daily routine with specific tasks for specific times. Time management and awareness of breaks/leisure activities play a major role.<br><br>**Anchor examples**: "Plan your day activities exactly", "Time management is the be-all and end-all. What people often forget, however, is to also plan for breaks, leisure activities, and also to stick to them just like working hours!"
**New habits**	All recommendations that propose the changing, creation and development of new habits are subsumed here. This may include the acceptance of the new routines brought by the pandemic as well the establishment of new practices that could be beneficial.<br><br>**Anchor examples:** „Changing Habits“, „new daily habits“, „adopt a new way of being“
**Setting goals**	This category includes recommendations linked to setting goals and objectives that may facilitate the way participants face and live through the pandemic period.<br><br>**Anchor examples**: „Create a focus, a daily goal, that shows them that each day is different from the last. I found this in writing the Diary“, „establish goals“, „aim to achieve objectives“
**Modesty and simplicity**	This category covers the recommendations to be modest and to limit oneself to essentials.<br><br>**Anchor examples:** „limit luxury“, „learn modesty“, „live simpler“

S2 Appendix. Themes and categories (T1 and T2).

**Adherence to containment measures**	This category includes the advice to adhere to COVID-19 containment measures coming from the government and to follow infection control recommendations.<br><br>**Anchor examples**: "adhere to government guidelines," "continue to wear masks when shopping and on public transportation, and keep your distance, as well as reasonable hygienic behaviour."
**Religion and spirituality**	Recommendations regarding religion and spirituality or religious/spiritual activities are included into this category.<br><br>**Anchor examples:** „prayer“, „engage with God“
**Being locally rooted**	This category contains recommendations related to regional living. Both the consumption of regional products as well as spending vacation in one’s own country are recorded here.<br><br>**Anchor examples:** „regional living“, „look out for regional products and spend vacation in Austria“
**Keeping contact with others**	This category includes the recommendations to keep up social contacts (family and friends) despite social restrictions, virtually if necessary. This includes cultivating contacts in general but also celebrating in virtual space, sharing worries and keeping contact with close people who are good for you.<br><br>**Anchor examples**: "Maintaining contacts virtually if necessary in order not to become socially wayward", "Talking to family/friends or even meeting in an appropriate setting if one has worries", "Keeping contacts".
**(Mutual) help/support**	This category includes the recommendations to support other people and also to seek help and/or accept help oneself when needed. This help includes also professional help.<br><br>**Anchor examples**: "Reaching out to others more and perhaps helping them", "Addressing needed help and accepting it yourself".
**Political recommendations**	This category includes statements of a political nature. When participants propose to change the government, speed up elections, vote for a specific political party, their answers are subsumed here.<br><br>**Anchor examples:** "Vote for... (specific party/politician)", "Change the Government", "Don't trust politicians!"
**Get vaccinated**	This category includes the recommendations to get vaccinated if possible, whatever the reason behind (own health, health of other people at risk, getting over with the pandemic etc.)<br><br>**Anchor examples:** “get vaccinated so that we sooner get out of the pandemic“, “get vaccinated so that we sooner get out of the pandemic”, “go get the vaccine”, “make sure you are fully vaccinated”

S2 Appendix. Themes and categories (T1 and T2).

**Other recommendations**	Recommendations that cannot be assigned to any other category are subsumed here. These are mostly very individual aspects that other participants would most likely not consider as recommendations for dealing with the pandemic.<br><br>Recommendations that were mentioned more than once are not subsumed here. If necessary, a separate category should be formed.
**No recommendations**	If participants indicated they had no recommendations, their responses are subsumed here.<br><br>**Anchor examples**: "None", "Unfortunately, I don't have an answer to that at the moment".
**Not specified**	If nothing was specified, this category is chosen.<br>Both "no indication", "n/a", "n/a" and any special characters (e.g. "/", "...") count as an answer for this category.

Trash

This **Rest-Category** includes all segments that are not understandable, as well as those that do not correspond to the definitions of the respective dimensions.

Example of incomprehensible segment: "Pointing out the almost helplessness of immediate solution".
Example of segment that does not meet the definition of the dimension: "Hope that you have a secure job and do not have to rely on others" (this is not a recommendation, but rather a comment)
