# Supplementary material for: Shaped by the COVID-19 pandemic: Psychological responses from a subjective perspective–A longitudinal mixed-methods study across five European countries
Source: PLoS One. 2023 Apr 25;18(4):e0285078. doi: 10.1371/journal.pone.0285078 (PMC10128933; doi:10.1371/journal.pone.0285078)
Supplement: S3 Appendix — (PDF) [file pone.0285078.s003.pdf]

S3 Appendix. Detailed results of the mixed-methods analysis.

Overview of themes

S1 Table. Themes related to stressful events during the pandemic.

	Austria T1	Austria T2	Croatia T1	Croatia T2	Georgia T1	Georgia T2	Greece T1	Greece T2	Portugal T1	Portugal T2
Restrictions and changes in daily life	33,0%	26,4%	29,9%	30,7%	26,4%	19,9%	20,8%	21,4%	18,3%	19,3%
COVID-19 and other health issues	4,4%	13,2%	3,9%	6,7%	12,5%	16,1%	19,4%	25,2%	12,7%	20,2%
Vaccination issues	0	3,7%	0	1,4%	0	3,7%	0	0,8%	0	0
Emotional distress	17,6%	13,7%	24,4%	20,7%	24,3%	23,0%	22,9%	22,1%	17,5%	14,3%
Work and finances	12,9%	10,9%	12,4%	8,9%	15,3%	9,9%	11,8%	6,1%	23,8%	16,8%
Burden related to loved ones	14,3%	15,3%	8,5%	10,4%	2,1%	8,7%	9,7%	12,2%	13,5%	12,6%
Societal impact	4,4%	3,9%	3,9%	4,3%	4,9%	5,0%	4,9%	2,3%	1,6%	1,7%
Pandemic management and communication	8,4%	8,8%	5,3%	11,8%	4,2%	12,4%	6,9%	3,8%	4,0%	9,2%
Other burden	3,0%	1,6%	10,3%	3,3%	5,6%	0,6%	1,4%	1,5%	6,3%	5,0%
No stressful events	1,9%	2,5%	1,4%	1,9%	4,9%	0,6%	2,1%	4,6%	2,4%	0,8%

S3 Appendix. Detailed results of the mixed-methods analysis.

S2 Table. Themes related to negative aspects of the pandemic.

	Austria T1	Austria T2	Croatia T1	Croatia T2	Georgia T1	Georgia T2	Greece T1	Greece T2	Portugal T1	Portugal T2
Restrictions and changes in daily life	22,5%	21,2%	15,1%	18,2%	34,8%	26,6%	21,2%	22,0%	41,2%	44,7%
COVID-19 and other health issues	7,9%	4,7%	10,2%	12,7%	6,5%	18,7%	21,8%	22,7%	7,0%	10,5%
Vaccination issues	0	9,1%	0	4,0%	0	2,2%	0	0,7%	0	0
Emotional distress	20,6%	17,2%	12,9%	11,9%	24,6%	23,0%	17,6%	23,3%	21,1%	18,4%
Work and finances	10,5%	4,2%	20,4%	8,4%	14,5%	8,6%	15,8%	5,3%	12,3%	8,8%
Burden related to loved ones	1,7%	3,2%	2,0%	2,6%	4,3%	3,6%	1,8%	0,7%	0	1,8%
Societal impact	18,9%	24,0%	15,3%	17,7%	2,9%	5,0%	9,1%	13,3%	8,8%	6,1%
Pandemic management and communication	12,9%	14,2%	14,9%	21,9%	7,2%	6,5%	9,1%	12,0%	7,9%	6,1%
Other negative aspects	3,9%	1,7%	8,3%	2,1%	3,6%	3,6%	3,6%	0	1,8%	3,5%
No negative aspects	1,1%	0,4%	0,8%	0,5%	1,4%	2,2%	0	0	0	0

S3 Appendix. Detailed results of the mixed-methods analysis.

S3 Table. Themes related to positive aspects of the pandemic.

	Austria T1	Austria T2	Croatia T1	Croatia T2	Georgia T1	Georgia T2	Greece T1	Greece T2	Portugal T1	Portugal T2
Reflection and growth	34,3%	25,8%	27,6%	35,7%	27,9%	24,6%	27,6%	41,5%	37,0%	36,2%
Opportunity for meaningful/enjoyable activities	19,7%	18,5%	16,8%	9,4%	24,0%	16,4%	20,9%	17,7%	8,3%	9,5%
Environmental effects	8,6%	7,1%	5,8%	2,8%	4,7%	0	1,5%	0,8%	2,8%	4,8%
Benefits on interpersonal level	17,4%	15,7%	22,9%	20,8%	18,6%	13,1%	17,9%	7,7%	18,5%	11,4%
Digitalisation and working/studying from home	6,5%	12,7%	9,7%	12,4%	10,1%	19,7%	9,7%	16,2%	16,7%	21,9%
Competent pandemic management	3,2%	2,3%	4,1%	2,0%	1,6%	0	5,2%	3,8%	1,9%	2,9%
Vaccination/Access to vaccination	0	4,3%	0	2,4%	0	3,3%	0	2,3%	0	1,0%
Other positive aspects	3,5%	3,8%	8,2%	4,2%	3,9%	10,7%	5,2%	0	8,3%	6,7%
No positive aspects	6,9%	9,9%	4,9%	10,4%	9,3%	12,3%	11,9%	10,0%	6,5%	5,7%

S3 Appendix. Detailed results of the mixed-methods analysis.

S4 Table. Themes related to recommendations how to cope with the pandemic.

	Austria T1	Austria T2	Croatia T1	Croatia T2	Georgia T1	Georgia T2	Greece T1	Greece T2	Portugal T1	Portugal T2
Beneficial behavioural adjustment	38,4%	35,6%	45,1%	44,9%	58,4%	43,5%	32,3%	28,3%	47,4%	48,5%
Beneficial cognitive-emotional strategies	40,2%	27,1%	23,1%	26,4%	23,2%	27,2%	48,8%	52,2%	28,4%	27,7%
Social support	15,2%	19,6%	16,0%	18,0%	12,8%	8,2%	14,0%	11,3%	13,8%	14,9%
Political recommendations	0	0,2%	0,2%	0,6%	0	0	0,6%	1,3%	0	1,0%
Get vaccinated	0	11,9%	0	3,9%	0	14,3%	0	3,1%	0	2,0%
Other recommendations	2,9%	1,5%	12,5%	2,2%	2,4%	2,7%	1,8%	0,6%	7,8%	2,0%
No recommendations	3,3%	4,1%	3,1%	3,9%	3,2%	4,1%	2,4%	3,1%	2,6%	4,0%

S3 Appendix. Detailed results of the mixed-methods analysis.

Comparison of themes based on participants' characteristics

S5 Table. Crosstabulation of sociodemographic characteristics and themes related to stressful events during the pandemic.

	Age	Gender			Relationship status		Living situation		Having children	
	M (*SD*)	Male	Female	Other	Single	In a relationship	Living alone	Living with other people	No	Yes
	N = 1070	*n* = 544	*n* = 1592	*n* = 4	*n* = 526	*n* = 1614	*n* = 568	*n* = 1572	*n* = 964	*n* = 1176
Restrictions and changes in daily life	43.3 (14.6)	27.2%	29.1%	40.0%	31.1%	27.9%	33.4%	27.0%	30.3%	27.3%
COVID-19 and other health issues	42.4 (13.5)	12.7%	9.6%	40.0%	9.6%	10.7%	9.3%	10.8%	10.9%	10.0%
Vaccination issues/ Imposed vaccination	42.1 (14.1)	1.4%	1.1%	0	1.2%	1.1%	0.7%	1.3%	0.9%	1.4%
Emotional distress	41.4 (12.0)	18.0%	21.8%	20.0%	20.7%	21.0%	20.1%	21.2%	21.6%	20.3%
Work and finances	40.2 (11.5)	12.6%	12.4%	0	12.1%	12.5%	11.2%	12.9%	13.4%	11.7%
Burden related to loved ones	41.4 (12.0)	8.4%	12.6%	0	10.6%	12.0%	12.5%	11.3%	9.8%	13.2%
Societal impact	42.4 (13.7)	5.2%	3.8%	0	4.3%	4.1%	3.3%	4.4%	4.6%	3.7%
Pandemic management and communication	46.6 (13.6)	11.9%	7.5%	0	7.4%	8.9%	7.2%	8.9%	6.8%	9.9%
No stressful events	46.3 (15.1)	2.5%	2.1%	0	3.1%	1.9%	2.3%	2.1%	1.8%	2.5%

S3 Appendix. Detailed results of the mixed-methods analysis.

S6 Table. Crosstabulation of sociodemographic characteristics and themes related to negative aspects of the pandemic.

	Age	Gender			Relationship status		Living situation		Having children	
	M (*SD*)	Male	Female	Other	Single	In a relationship	Living alone	Living with other people	No	Yes
	N = 1070	*n* = 544	*n* = 1592	*n* = 4	*n* = 526	*n* = 1614	*n* = 568	*n* = 1572	*n* = 964	*n* = 1176
Restrictions and changes in daily life	41.9 (13.5)	27.2%	29.1%	40.0%	31.1%	27.9%	33.4%	27.0%	30.3%	27.3%
COVID-19 and other health issues	49.0 (14.7)	22.2%	23.3%	20.0%	25.9%	22.1%	26.4%	21.9%	25.2%	21.1%
Vaccination issues/ Imposed vaccination	41.4 (13.0)	3.0%	2.4%	0	2.6%	2.5%	2.9%	2.4%	1.9%	3.1%
Emotional distress	41.6 (12.4)	12.4%	10.9%	20.0%	11.6%	11.2%	11.4%	11.2%	12.8%	10.0%
Work and finances	41.1 (12.7)	13.8%	18.8%	20.0%	19.7%	16.9%	16.3%	18.0%	17.4%	17.8%
Burden related to loved ones	40.5 (11.3)	11.2%	11.7%	0	11.4%	11.7%	11.0%	11.8%	12.3%	11.0%
Societal impact	43.8 (13.8)	1.3%	2.7%	0	2.0%	2.5%	1.4%	2.8%	1.4%	3.2%
Pandemic management and communication	44.3 (13.3)	17.6%	15.7%	40.0%	14.2%	16.9%	16.4%	16.1%	14.9%	17.4%
No negative aspects	45.3 (14.1)	18.0%	13.6%	0	11.4%	15.7%	14.0%	14.9%	13.5%	15.7%

S3 Appendix. Detailed results of the mixed-methods analysis.

S7 Table. Crosstabulation of sociodemographic characteristics and themes related to positive aspects of the pandemic.

	Age	Gender			Relationship status		Living situation		Having children	
	M (*SD*)	Male	Female	Other	Single	In a relationship	Living alone	Living with other people	No	Yes
	N = 1070	*n* = 544	*n* = 1592	*n* = 4	*n* = 526	*n* = 1614	*n* = 568	*n* = 1572	*n* = 964	*n* = 1176
Reflection and growth	42.8 (13.2)	32.4%	33.3%	28.6%	35.8%	32.2%	34.2%	32.7%	34.8%	31.6%
Opportunity for meaningful /enjoyable activities	39.6 (12.9)	15.1%	17.6%	0	17.0%	16.9%	15.0%	17.6%	20.8%	13.7%
Environmental effects	44.4 (12.4)	6.6%	4.7%	14.3%	5.1%	5.2%	5.6%	5.0%	5.0%	5.3%
Benefits on interpersonal level	43.1 (13.4)	14.4%	20.5%	42.9%	15.9%	20.2%	17.2%	19.8%	15.3%	22.3%
Digitalisation and working /studying from home	40.0 (11.8)	13.7%	12.0%	14.3%	11.8%	12.6%	12.8%	12.2%	13.2%	11.7%
Competent pandemic management	50.1 (15.7)	5.2%	2.4%	0	2.7%	3.1%	3.9%	2.7%	2.7%	3.3%
Vaccination/Access to vaccination	55.5 (14.2)	2.4%	1.2%	0	2.2%	1.3%	2.8%	1.0%	0.8%	2.1%
No positive aspects	45.3 (13.6)	10.4%	8.3%	0	9.6%	8.5%	8.5%	8.9%	7.5%	9.9%

S3 Appendix. Detailed results of the mixed-methods analysis.

S8 Table. Crosstabulation of sociodemographic characteristics and themes related to recommendations for dealing with the pandemic.

	Age	Gender			Relationship status		Living situation		Having children	
	M (*SD*)	Male	Female	Other	Single	In a relationship	Living alone	Living with other people	No	Yes
	N = 1070	*n* = 544	*n* = 1592	*n* = 4	*n* = 526	*n* = 1614	*n* = 568	*n* = 1572	*n* = 964	*n* = 1176
Beneficial behavioural adjustment	43.3 (13.6)	43.6%	43.2%	66.7%	42.8%	43.5%	41.8%	43.9%	43.7%	43.0%
Beneficial cognitive-emotional strategies	43.3 (13.3)	35.3%	31.3%	0	32.5%	32.2%	33.0%	32.0%	30.1%	34.1%
Social support	40.9 (13.5)	11.9%	18.0%	33.3%	16.1%	16.6%	15.8%	16.7%	19.0%	14.3%
Political recommendations	39.4 (6.9)	0.8%	0.2%	0	0.4%	0.3%	0.5%	0.3%	0.5%	0.2%
Get vaccinated	49.8 (15.4)	4.5%	3.7%	0	4.6%	3.7%	4.3%	3.8%	3.0%	4.7%
Do not get vaccinated	36.0 (0.0)	0.2%	0	0	0	0.0%	0.1%	0	0.1%	0
No recommendations	44.3 (13.4)	3.8%	3.6%	0	3.6%	3.7%	4.5%	3.3%	3.6%	3.7%

S3 Appendix. Detailed results of the mixed-methods analysis.

S9 Table. Stressful events in relation to health-related characteristics, social factors and financial situation.

	Restrictions and changes in daily life *n* = 777	COVID-19 and other health issues *n* = 282	Vaccination issues *n* = 31	Emotional distress *n* = 566	Work and finances *n* = 337	Burden related to loved ones *n* = 315	Societal impact *n* = 112	Pandemic management and communication *n* = 230	No stressful events *n* = 59
	M (*SD*)	*M* (*SD*)	*M* (*SD*)	*M* (*SD*)	*M* (*SD*)	*M* (*SD*)	*M* (*SD*)	*M* (*SD*)	*M* (*SD*)
T1: Health status	1.9 (0.8)	1.9 (0.8)	1.8 (0.9)	2.0 (0.9)	2.0 (0.8)	1.9 (0.9)	1.9 (0.8)	1.9 (0.8)	1.7 (0.7)
T2: Health status	2.0 (0.9)	2.1 (0.9)	1.8 (0.7)	2.1 (0.9)	2.1 (0.9)	2.1 (0.9)	2.0 (0.8)	2.1 (0.8)	1.9 (0.9)
T1: Face-to-face contact to loved ones	2.3 (1.3)	2.1 (1.4)	2.6 (1.4)	1.9 (1.4)	1.9 (1.4)	2.0 (1.4)	2.1 (1.4)	2.2 (1.4)	2.3 (1.4)
T2: Face-to-face contact to loved ones	2.8 (1.2)	2.6 (1.2)	2.9 (1.2)	2.6 (1.2)	2.6 (1.2)	2.7 (1.1)	2.7 (1.2)	2.8 (1.2)	2.6 (1.3)
T1: Virtual contact to loved ones	4.0 (1.3)	4.0 (1.2)	3.7 (1.2)	4.0 (1.3)	3.9 (1.3)	3.9 (1.3)	4.0 (1.3)	3.9 (1.4)	3.7 (1.4)
T2: Virtual contact to loved ones	3.5 (1.5)	3.7 (1.4)	3.7 (1.5)	3.5 (1.5)	3.5 (1.4)	3.5 (1.4)	3.5 (1.5)	3.4 (1.6)	3.6 (1.3)
	%	%	%	%	%	%	%	%	%
T1: COVID-19 infection	0.4	1.4	0	0.4	0.6	1.0	0	0.4	0
T2: COVID-19 infection	14.2	22.7	6.5	19.3	19.6	14.9	14.3	18.3	25.4
T1: Risk for severe course of COVID-19	19.8	22.7	22.6	21.4	16.6	16.8	19.6	24.8	18.6
T2: Risk for severe course of COVID-19	15.1	14.9	6.5	17.5	14.2	12.7	19.6	15.2	6.8
T1: More time at home	89.5	93.4	72.4	93.8	86.0	90.6	84.3	84.0	77.8
T2: More time at home	69.0	73.5	43.3	72.3	68.3	66.0	67.6	63.8	58.8
T1: Pandemic-related income loss	35.6	32.6	29.0	40.1	43.9	30.5	36.6	28.3	25.4
T2: Pandemic-related income loss	22.8	23.0	22.6	25.3	30.3	21.9	19.6	21.7	6.8

S3 Appendix. Detailed results of the mixed-methods analysis.

S10 Table. Negative aspects of the pandemic in relation to health-related characteristics, social factors and financial situation.

	Restrictions and changes in daily life *n* = 655	COVID-19 and other health issues *n* = 321	Vaccination issues *n* = 72	Emotional distress *n* = 500	Work and finances *n* = 330	Burden related to loved ones *n* = 68	Societal impact *n* = 461	Pandemic management and communication *n* = 416	No negative aspects *n* = 20
	M (*SD*)	*M* (*SD*)	*M* (*SD*)	*M* (*SD*)	*M* (*SD*)	*M* (*SD*)	*M* (*SD*)	*M* (*SD*)	*M* (*SD*)
T1: Health status	2.0 (0.8)	1.9 (0.8)	1.8 (0.7)	2.1 (0.9)	1.9 (0.8)	1.9 (0.8)	1.9 (0.8)	1.8 (0.8)	1.9 (0.7)
T2: Health status	2.1 (0.8)	2.0 (0.9)	1.9 (0.8)	2.1 (0.9)	2.0 (0.9)	2.0 (0.8)	2.0 (0.9)	2.0 (0.9)	1.5 (0.6)
T1: Face-to-face contact to loved ones	2.1 (1.4)	2.1 (1.4)	2.4 (1.3)	2.2 (1.4)	2.0 (1.3)	2.1 (1.3)	2.3 (1.3)	2.2 (1.4)	2.2 (1.2)
T2: Face-to-face contact to loved ones	2.8 (1.1)	2.6 (1.2)	3.0 (1.0)	2.7 (1.2)	2.6 (1.2)	2.6 (1.1)	2.8 (1.2)	2.7 (1.2)	2.8 (1.3)
T1: Virtual contact to loved ones	4.0 (1.2)	4.0 (1.4)	4.1 (1.1)	4.0 (1.3)	3.9 (1.3)	4.0 (1.4)	3.8 (1.4)	3.8 (1.4)	4.5 (0.9)
T2: Virtual contact to loved ones	3.6 (1.5)	3.6 (1.5)	3.8 (1.3)	3.5 (1.5)	3.5 (1.5)	3.6 (1.3)	3.4 (1.5)	3.4 (1.5)	3.7 (1.3)
	%	%	%	%	%	%	%	%	%
T1: COVID-19 infection	0.6	0.6	1.4	0.4	0	0	0.4	1.0	0
T2: COVID-19 infection	15.9	15.9	18.1	18.6	19.7	20.6	12.1	17.5	35.0
T1: Risk for severe course of COVID-19	20.8	20.9	16.7	19.4	16.4	20.6	20.6	17.3	30.0
T2: Risk for severe course of COVID-19	13.1	18.1	11.1	14.6	11.5	14.7	16.9	15.4	5.0
T1: More time at home	89.7	96.7	80.0	91.5	89.9	92.2	84.9	85.3	84.2
T2: More time at home	67.7	82.1	59.4	64.1	75.3	74.1	65.0	61.3	55.0
T1: Pandemic-related income loss	34.7	40.5	23.6	38.8	45.2	14.2	30.2	32.9	20.0
T2: Pandemic-related income loss	22.3	24.0	16.7	25.4	31.2	25.0	20.2	21.6	10.0

S3 Appendix. Detailed results of the mixed-methods analysis.

S11 Table. Positive aspects of the pandemic in relation to health-related characteristics, social factors and financial situation.

	Reflection and growth	Opportunity for meaningful/enjoy-able activities	Environmental effects	Benefits on interpersonal level	Digitalisation and working/ studying from home	Competent pandemic management	Vaccination /Access to vaccination	No positive aspects
	n = 812	*n* = 416	*n* = 127	*n* = 470	*n* = 304	*n* = 74	*n* = 37	*n* = 216
	M (*SD*)	*M* (*SD*)	*M* (*SD*)	*M* (*SD*)	*M* (*SD*)	*M* (*SD*)	*M* (*SD*)	*M* (*SD*)
T1: Health status	1.9 (0.8)	1.8 (0.8)	1.8 (0.8)	1.9 (0.8)	1.9 (0.8)	1.9 (0.8)	1.9 (0.8)	2.0 (0.9)
T2: Health status	2.0 (0.9)	2.0 (0.9)	2.1 (0.9)	2.1 (0.8)	2.0 (0.8)	2.1 (0.8)	2.0 (0.9)	2.1 (0.9)
T1: Face-to-face contact to loved ones	2.1 (1.4)	2.2 (1.3)	2.4 (1.3)	2.1 (1.4)	1.9 (1.4)	2.3 (1.3)	2.3 (1.4)	2.3 (1.4)
T2: Face-to-face contact to loved ones	2.7 (1.1)	2.8 (1.1)	2.8 (1.1)	2.6 (1.2)	2.6 (1.2)	2.6 (1.1)	2.6 (1.0)	2.7 (1.2)
T1: Virtual contact to loved ones	3.9 (1.3)	3.8 (1.4)	3.8 (1.3)	4.1 (1.3)	4.0 (1.2)	3.8 (1.3)	4.2 (1.2)	4.0 (1.3)
T2: Virtual contact to loved ones	3.5 (1.5)	3.4 (1.5)	3.4 (1.5)	3.5 (1.5)	3.7 (1.3)	3.7 (1.3)	3.9 (1.2)	3.6 (1.5)
	%	%	%	%	%	%	%	%
T1: COVID-19 infection	0.5	0.2	0.8	0.2	1.0	1.4	0	0.9
T2: COVID-19 infection	16.1	14.4	15.7	17.0	19.4	9.5	16.2	17.1
T1: Risk for severe course of COVID-19	19.0	17.5	16.5	20.4	15.1	35.1	32.4	19.0
T2: Risk for severe course of COVID-19	14.4	11.1	12.6	16.8	12.8	25.7	13.5	14.8
T1: More time at home	88.7	91.3	87.0	88.7	95.3	89.0	94.4	81.4
T2: More time at home	68.9	66.3	59.3	68.8	71.9	82.9	76.5	61.0
T1: Pandemic-related income loss	32.8	38.9	35.4	37.2	34.9	25.7	29.7	33.8
T2: Pandemic-related income loss	20.3	21.9	26.8	22.6	17.8	23.0	29.7	28.7

S3 Appendix. Detailed results of the mixed-methods analysis.

S12 Table. Recommendations for dealing with the pandemic in relation to health-related characteristics, social factors and financial situation.

	Beneficial behavioural adjustment *n* = 1164	Beneficial cognitive-emotional strategies *n* = 867	Social support *n* = 443	Political recommendations *n* = 9	Get vaccinated *n* = 105	No recommendations *n* = 98
	M (*SD*)	*M* (*SD*)	*M* (*SD*)	*M* (*SD*)	*M* (*SD*)	*M* (*SD*)
T1: Health status	1.9 (0.8)	1.8 (0.8)	2.0 (0.8)	1.8 (0.8)	2.0 (0.9)	2.1 (0.8)
T2: Health status	2.0 (0.9)	1.9 (0.8)	2.1 (0.9)	2.1 (0.7)	2.2 (0.9)	2.3 (0.9)
T1: Face-to-face contact to loved ones	2.2 (1.4)	2.3 (1.3)	2.1 (1.4)	2.2 (1.4)	2.2 (1.4)	1.9 (1.3)
T2: Face-to-face contact to loved ones	2.7 (1.2)	2.7 (1.2)	2.8 (1.2)	2.8 (1.0)	2.8 (1.1)	2.4 (1.2)
T1: Virtual contact to loved ones	4.0 (1.3)	4.0 (1.3)	3.9 (1.3)	3.3 (1.4)	4.0 (1.3)	3.7 (1.4)
T2: Virtual contact to loved ones	3.5 (1.5)	3.5 (1.5)	3.4 (1.5)	3.6 (1.3)	3.9 (1.4)	3.2 (1.6)
	%	%	%	%	%	%
T1: COVID-19 infection	0.5	0.7	1.1	0	0	0
T2: COVID-19 infection	16.4	14.2	18.7	11.1	12.4	15.3
T1: Risk for severe course of COVID-19	19.8	19.7	18.1	22.2	33.3	22.4
T2: Risk for severe course of COVID-19	13.8	12.6	13.5	22.2	21.0	26.5
T1: More time at home	89.6	88.0	91.9	100	81.9	84.4
T2: More time at home	68.5	68.3	67.5	77.8	62.9	71.7
T1: Pandemic-related income loss	34.9	34.3	33.6	33.3	24.8	34.7
T2: Pandemic-related income loss	20.3	24.0	22.3	22.2	17.1	28.6
