# Supplementary material for: Shaped by the COVID-19 pandemic: Psychological responses from a subjective perspective–A longitudinal mixed-methods study across five European countries
Source: PLoS One. 2023 Apr 25;18(4):e0285078. doi: 10.1371/journal.pone.0285078 (PMC10128933; doi:10.1371/journal.pone.0285078)
Supplement: Attachment — Submitted filename: Response to Reviewers.pdf [file pone.0285078.s004.pdf]

PONE-D-22-35029
Shaped by the COVID-19 pandemic: Psychological responses from a subjective perspective – A longitudinal mixed-methods study across five European countries
PLOS ONE

We would like to thank the academic editor and both reviewers for taking the time to review our manuscript. We highly appreciate the positive comments and the valuable suggestions, which have certainly helped us to further improve the quality of the manuscript.

In the following, please find our responses to the comments raised by the editor and the reviewers:

<u>Response to the academic editor</u>

1. Please ensure that your manuscript meets PLOS ONE's style requirements, including those for file naming. The PLOS ONE style templates can be found at [*https://journals.plos.org/plosone/s/file?id=wjVg/PLOSOne_formatting_sample_main_body.pdf*](https://journals.plos.org/plosone/s/file?id=wjVg/PLOSOne_formatting_sample_main_body.pdf) *and* [*https://journals.plos.org/plosone/s/file?id=ba62/PLOSOne_formatting_sample_title_authors_affiliations.pdf*](https://journals.plos.org/plosone/s/file?id=ba62/PLOSOne_formatting_sample_title_authors_affiliations.pdf)

Response: The paper has been revised to meet PLOS ONE's style requirements and the file names have been changed accordingly. Moreover, all figures were adapted using the PACE digital diagnostic tool.

2. Please ensure that you have specified (1) whether consent was informed and (2) what type you obtained (for instance, written or verbal, and if verbal, how it was documented and witnessed). If your study included minors, state whether you obtained consent from parents or guardians. If the need for consent was waived by the ethics committee, please include this information.

Response: We have now specified that written informed consent was obtained from the participants:

"After receiving information about the study aims, data management, confidentiality, and right to withdraw, all participants provided written informed consent." (Methods, page 8)

3. We note that the grant information you provided in the 'Funding Information' and 'Financial Disclosure' sections do not match. When you resubmit, please ensure that you provide the correct grant numbers for the awards you received for your study in the 'Funding Information' section.

Response: Thank you for pointing out that the information in the 'Funding Information' and 'Financial Disclosure' sections did not match. As stated in the 'Financial Disclosure' in the manuscript, we did not receive any funding for conducting the study, only open access funding will be provided by the University of Vienna, in case the manuscript will be accepted. Thus, the finals statement reads as follows:

"The authors received no specific funding for conducting the study. Open access funding provided by the University of Vienna."

4. In your Data Availability statement, you have not specified where the minimal data set underlying the results described in your manuscript can be found. PLOS defines a study's minimal data set as the underlying data used to reach the conclusions drawn in the manuscript and any additional data required to replicate the reported study findings in their entirety. All PLOS journals require that the

minimal data set be made fully available. For more information about our data policy, please see http://journals.plos.org/plosone/s/data-availability.

Upon re-submitting your revised manuscript, please upload your study's minimal underlying data set as either Supporting Information files or to a stable, public repository and include the relevant URLs, DOIs, or accession numbers within your revised cover letter. For a list of acceptable repositories, please see http://journals.plos.org/plosone/s/data-availability#loc-recommended-repositories. Any potentially identifying patient information must be fully anonymized.

Important: If there are ethical or legal restrictions to sharing your data publicly, please explain these restrictions in detail. Please see our guidelines for more information on what we consider unacceptable restrictions to publicly sharing data: http://journals.plos.org/plosone/s/data-availability#loc-unacceptable-data-access-restrictions. Note that it is not acceptable for the authors to be the sole named individuals responsible for ensuring data access.

We will update your Data Availability statement to reflect the information you provide in your cover letter.

Response: Thank you for reminding us of this important point. The minimal data set underlying the quantitative results is made fully available, along with the experts of the qualitative data relevant to the study. All data can be found in the OSF repository under this link: https://osf.io/p9jx2/; doi: 10.17605/OSF.IO/P9JX2.

5. We note that you have stated that you will provide repository information for your data at acceptance. Should your manuscript be accepted for publication, we will hold it until you provide the relevant accession numbers or DOIs necessary to access your data. If you wish to make changes to your Data Availability statement, please describe these changes in your cover letter and we will update your Data Availability statement to reflect the information you provide.

Response: As stated earlier, the study's minimal underlying data set and the supporting information will be deposited to a public repository. The data can be accessed under: https://osf.io/p9jx2/

6. Please include your full ethics statement in the 'Methods' section of your manuscript file. In your statement, please include the full name of the IRB or ethics committee who approved or waived your study, as well as whether or not you obtained informed written or verbal consent. If consent was waived for your study, please include this information in your statement as well.

Response: All details regarding the ethical approval are now provided in the 'Methods' section and removed from the supplementary material. The ethics statement in the revised version reads as follows:

"After receiving information about the study aims, data management, confidentiality, and right to withdraw, all participants provided written informed consent. Ethical approval was obtained in all participating countries:
Austria: Ethics Committee of the University of Vienna, Reference Number: 00554
Croatia: Ethics Committee of the Department of Psychology, Faculty of Humanities and Social Sciences, University of Zagreb: 21/05/2020
Georgia: Ilia State University, Faculty of Arts and Science, Research Ethics Committee: 12/06/2020
Greece: Social Sciences Ethics Review Board (SSERB), University of Nicosia: SSERB 00109
Portugal: Ethics Committee of the Medical Faculty, University of Porto and Centro Hospitalar São João, Porto, Portugal: CE 201-20"

7. Please include captions for your Supporting Information files at the end of your manuscript, and update any in-text citations to match accordingly. Please see our Supporting Information guidelines for more information: http://journals.plos.org/plosone/s/supporting-information.

Response: The in-text citations referring to the supplemental material have been updated. We also added the captions for our Supporting Information at the end of the revised manuscript:

S1 Appendix. Detailed information on the methodology and participants' characteristics.

S2 Appendix. Themes and categories (T1 and T2).

S3 Appendix. Detailed results of the mixed-methods analysis.

8. Please review your reference list to ensure that it is complete and correct. If you have cited papers that have been retracted, please include the rationale for doing so in the manuscript text, or remove these references and replace them with relevant current references. Any changes to the reference list should be mentioned in the rebuttal letter that accompanies your revised manuscript. If you need to cite a retracted article, indicate the article's retracted status in the References list and also include a citation and full reference for the retraction notice.

Response: The reference list has been reviewed and in-text citations revised.

Response to the reviewers

Reviewer #1: The manuscript contains mixed qualitative and quantitative comparisons of adult mental health in five European countries in a longitudinal study design. The introduction is concise, but exhaustive. The method is well described and allows the replication of these studies. Statistical tests are appropriate for the study hypothesis verification. However, suggest to include effect size for all Student's t-tests, Mann-Whitney's U-tests and Pearson's chi-square tests in the manuscript (see pages 15-16, and Table 3). Figures and tables are helpful, as well as supplementary materials. Discussion and conclusion are well-written and adequate to the data found in the study. Overall, I highly appreciate the content, analyses and hard work to compare five countries in a such large number quantitative and qualitative data.

Response: We would like to thank the reviewer for the positive evaluation of the manuscript. We highly appreciate it. The suggestion to include effect size is very helpful. In the revised version of the manuscript, the appropriate measures of effect size are provided on pages 15-16 as well as in Table 3. Furthermore, we added a sentence on effect size measures in the 'Data analysis' section, which reads as follows:

"Depending on the analysis, different measures of effect size (Cohen's *d*, Odds ratio [OR], Rosenthal's *r*, Omega squared [ω2]) were reported."

Reviewer #2: This is a valid study and really helps to understand for clinicians how to navigate therapy and the understandings of the pandemic. I would have loved to see a section of the paper that focusses on post traumatic growth as explaining why not all people were affected negatively and also to show a link between how stressful events can foster resilience.
I did not see a distribution of how many people were involved per country, i think that would be a valuable addition.

Response: We very much appreciate this positive evaluation of our manuscript and thank the reviewer for the compliments and the helpful suggestions. The number of people per country can be found in Table 1 on page 9, along with relevant sociodemographic characteristics for each of the participating countries. We hope that this table contains all information that you consider necessary. Further details on participants' characteristics per country are provided in S1 Appendix. The idea to include a section on posttraumatic growth is very valuable and we thank you for proposing it. In the subchapter on positive aspects in the 'Discussion' section, we now touch upon posttraumatic growth in the following manner:

"*Reflection and growth* was the most prominent positive theme at both time points. Participants positively evaluated the increased appreciation for life and the opportunity to rethink their priorities. Such new perspectives on life have often been discussed in the context of posttraumatic growth (i.e. positive psychological changes following an adverse life event; [84]). There is some evidence suggesting that perceptions of growth could reflect coping efforts during the COVID-19 pandemic [85]. Although perceived growth might signalise resilience, it might also signalise greater posttraumatic stress symptoms [86, 87], which supports the idea of high variability in individual responses to stressful events. Regarding variability in our study, a lower number of people reported individual or community growth at T2 than at T1 in Austria."
